# Efficacy of soft tissue augmentation in the maxillary esthetic region: A 5‐year randomized controlled trial

**DOI:** 10.1002/JPER.24-0660

**Published:** 2025-05-19

**Authors:** Elise G. Zuiderveld, Gerry M. Raghoebar, Arjan Vissink, Barzi Gareb, Henny J. A. Meijer

**Affiliations:** ^1^ Department of Oral and Maxillofacial Surgery University Medical Center Groningen University of Groningen Groningen The Netherlands; ^2^ Department of Restorative Dentistry Dental School, University Medical Center Groningen University of Groningen Groningen The Netherlands

**Keywords:** alveolar bone loss, bone transplantation, dental implants, esthetics, soft tissue therapy

## Abstract

**Background:**

Soft tissue grafting at dental implant sites has been proposed to enhance esthetic outcomes. A xenogeneic collagen matrix (XCM) was introduced as an alternative grafting material to connective tissue. Only short‐term results are yet available.

**Methods:**

Sixty patients were treated in a randomized controlled trial with a connective tissue graft (*n* = 20, CTG group), an XCM (*n* = 20, XCM group), or received no graft (*n* = 20, NG group). The grafts were placed at the time of implant placement in a preserved alveolar ridge. The primary outcome was a change in mid‐buccal mucosal level (MBML) after 5 years (T_60_). Secondary outcomes were marginal bone level, clinical peri‐implant parameters, esthetics, and patient satisfaction.

**Results:**

At T_60_, mean changes in MBML were −0.41 ± 1.20 mm, −0.30 ± 1.22 mm, and −0.61 ± 1.72 mm in the CTG, XCM, and NG groups (*p* = 0.78), respectively. Also, regarding the secondary outcome variables, no significant between‐group differences were observed.

**Conclusions:**

Soft tissue grafting at single implant placement after alveolar ridge preservation, either with a CTG or  XCM, does not result in a better esthetic outcome and should not be considered as a standard procedure.

**Plain language summary:**

Implant placement in case of a failing tooth is a favorable treatment option. However, since the extraction socket is often associated with a large bone defect, alveolar ridge preservation with bone grafts prior to implant placement is often needed. To compensate for possible soft tissue defects, the application of a CTG or an XCM has been proposed. The question has arisen whether the use of an XCM will give a better outcome than a CTG. Furthermore, are both soft tissue augmentation therapies accompanied by a better esthetic result than performing no soft tissue therapy at all? Therefore, a 5‐year study was carried out in which 60 patients with a failing tooth in the frontal region of the upper jaw were treated with removal of the tooth and restoring the gap with bone graft and sealing the socket with mucosagraft from the tuberosity region. At the time of implant placement 5 months thereafter, 20 patients received a CTG, 20 patients received an XCM, and 20 patients no soft tissue therapy. After 5 years, it appeared that there was no difference between the 3 soft tissue treatment procedures. Thus, implant placement combined with soft tissue grafting in preserved alveolar ridges does not result in a better esthetic outcome.

## 1 INTRODUCTION

Single implant treatment in the maxillofacial esthetic region is a successful treatment option with satisfied patients.^1^, ^2^ Prerequisite to achieve good esthetics and stability of the result is a correct 3‐dimensional implant position with sufficient buccal bone for a soft tissue support.^3^, ^4^ Although it has been recognized that immediate implant placement gives comparable clinical outcomes to early and delayed placement and patients prefer it, this treatment option is not always possible due to insufficient remaining bone volume after tooth removal.^5^, ^6^, ^7^, ^8^ Augmentation of the extraction socket is needed in these cases to preserve and restore the alveolar ridge and buccal soft tissue.^9^, ^10^ Some bone loss as well as some soft tissue changes, both in width and height, still occur despite alveolar ridge preservation, however.^11^

It has been stated that soft tissue volume can be increased with a connective tissue graft (CTG) and hereby establishes a better soft tissue profile.^12^, ^13^, ^14^ A CTG has long time been seen as the golden standard, although some morbidity caused by the harvesting procedure was recognized.^15^, ^16^ A xenogeneic collagen matrix (XCM) was introduced to decrease this morbidity.^17^, ^18^ In the systematic review of Stefanini et al.,^14^ it was concluded that implants receiving a soft tissue augmentation exhibited in general favorable outcomes with a stable soft tissue margin over time, while implants without a soft tissue augmentation tend to exhibit an apical shift of the soft tissue margin in the medium and long term. However, it was also mentioned in this systematic review that randomized controlled trials (RCTs) with a longer follow‐up period are scarce and are needed to perform robust statistical analyses.

Prospective comparative studies of at least 5 years (either or not randomized) on soft tissue augmentation with CTG or XCM at implant sites are limited to Cosyn et al.,^19^ Fenner et al.,^20^ Hosseini et al.,^21^ Thoma et al.,^22^ and Zuiderveld et al.^23^ Outcome measures are expressed by mid‐buccal mucosa level, mucosa thickness, and ridge contour change. No long‐term studies of at least 10 years could be found on this subject.

Cosyn et al.^19^ reported the results of a 5‐year non‐randomized prospective study with 22 patients in which the application of a CTG at implant sites was compared with the use of no graft (NG). Mid‐buccal mucosa level change was −0.50 mm in the CTG group and −0.63 mm in the NG group, meaning recession. Fenner et al.^20^ reported on an 8‐year non‐randomized prospective study with 28 patients in which the application of a CTG at implant sites was compared with the use of NG. Mid‐buccal mucosa level change was −0.50 mm in the CTG group and +0.23 mm in the NG group, without a significant difference. Hosseini et al.^21^ reported on a 5‐year non‐randomized prospective study with 19 patients in which the application of a CTG at implant sites was compared with the use of NG. Mid‐buccal mucosa level change was +0.71 mm in the CTG group and +0.16 mm in the NG group, with a significant difference between the groups. Next to this, a significant difference in mucosa thickness (a higher thickness in the CTG group) was reported. Thoma et al.^22^ reported on a 5‐year randomized controlled trial with 20 patients in which the application of a CTG at implant sites was compared with the application of an XCM. They reported no significant difference in ridge contour change between the groups. Zuiderveld et al.^23^ reported on a 5‐year randomized controlled trial with 60 patients in which the application of a CTG at implant sites was compared with the use of NG. Mid‐buccal mucosa level change was +0.10 mm in the CTG group and −0.60 mm in the NG group, with a significant difference between the groups. None of these trials compared the soft tissue augmentation procedures with each other and with the absence of an augmentation procedure. Therefore, the aim of the present 5‐year 3‐arm RCT was to compare mid‐buccal mucosa changes after grafting the implant site with either a CTG or XCM compared to no grafting at implant placement in a preserved alveolar ridge in the maxillary esthetic region.

## 2 MATERIALS AND METHODS

### 2.1 Study design

The initial 1‐year study was set up as an RCT.^24^ The 1‐year study was approved by the Medical Ethical Committee (NL43085.042.13) and registered in the Netherlands Trial Register (NTR3815; date 01‐23‐2013). The Medical Ethical Committee concluded that the 5‐year follow‐up was not a new clinical research, but part of a regular control appointment without the collection of additional data (METc communication M21.285739, date November 3, 2021). The 5‐year study was also registered in the Netherlands Trial Register (NTR9860 (date 11‐5‐2021). All patients gave written informed consent with the 5‐year study and verbally approved the use of the research data.

Between December 2012 and July 2015, consecutive patients with a single failing maxillary tooth in the esthetic region and not enough bone for immediate implant placement were asked to take part in an RCT. After approval, the failing tooth was removed and the extraction socket was augmented and closed with a mucosa graft. After 4 months, a dental implant was placed and thereafter the patients were randomly distributed to receive either:

1. no soft tissue graft (NG group; *n* = 20);
2. a CTG harvested from the palate (CTG group; *n* = 20);
3. an XCM (XCM group, *n* = 20).

Details of the study design and 1‐year follow‐up results have been described previously.^24^

### 2.2 Intervention procedure

One day prior to implant surgery, patients started taking antibiotics (amoxicillin 500 mg, t.i.d. for 7 days or clindamycin 300 mg, q.i.d. for 7 days in case of amoxicillin allergy) and used a 0.2% chlorhexidine mouthwash (twice daily for 7 days) for oral disinfection. In all groups, the extraction socket was augmented with the tuberosity bone graft shaped to match the buccal bone defect and inserted with the cortical side facing the periosteum. A mixture of autologous bone and spongious bone substitute (0.25–1.0 mm)* was tightly packed into the extraction socket. Then, the extraction socket was closed with a full‐thickness epithelialized gingival graft, which was also harvested from the maxillary tuberosity region.

**FIGURE 1:**
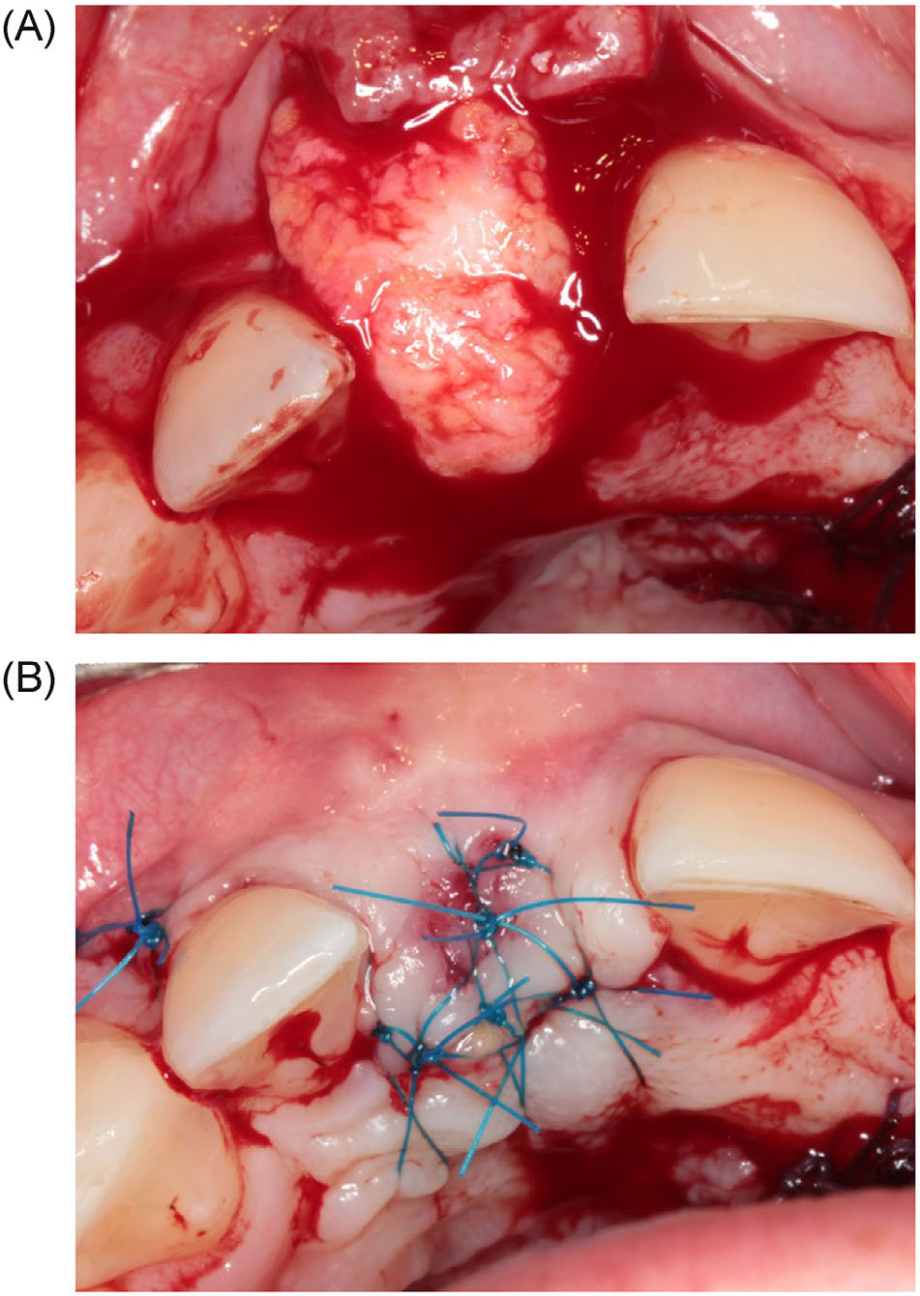
(A) Placement of the CTG in the prepared mucoperiosteal envelope. (B) CTG secured with horizontal and vertical mattress sutures. CTG, connective tissue graft.

**FIGURE 2:**
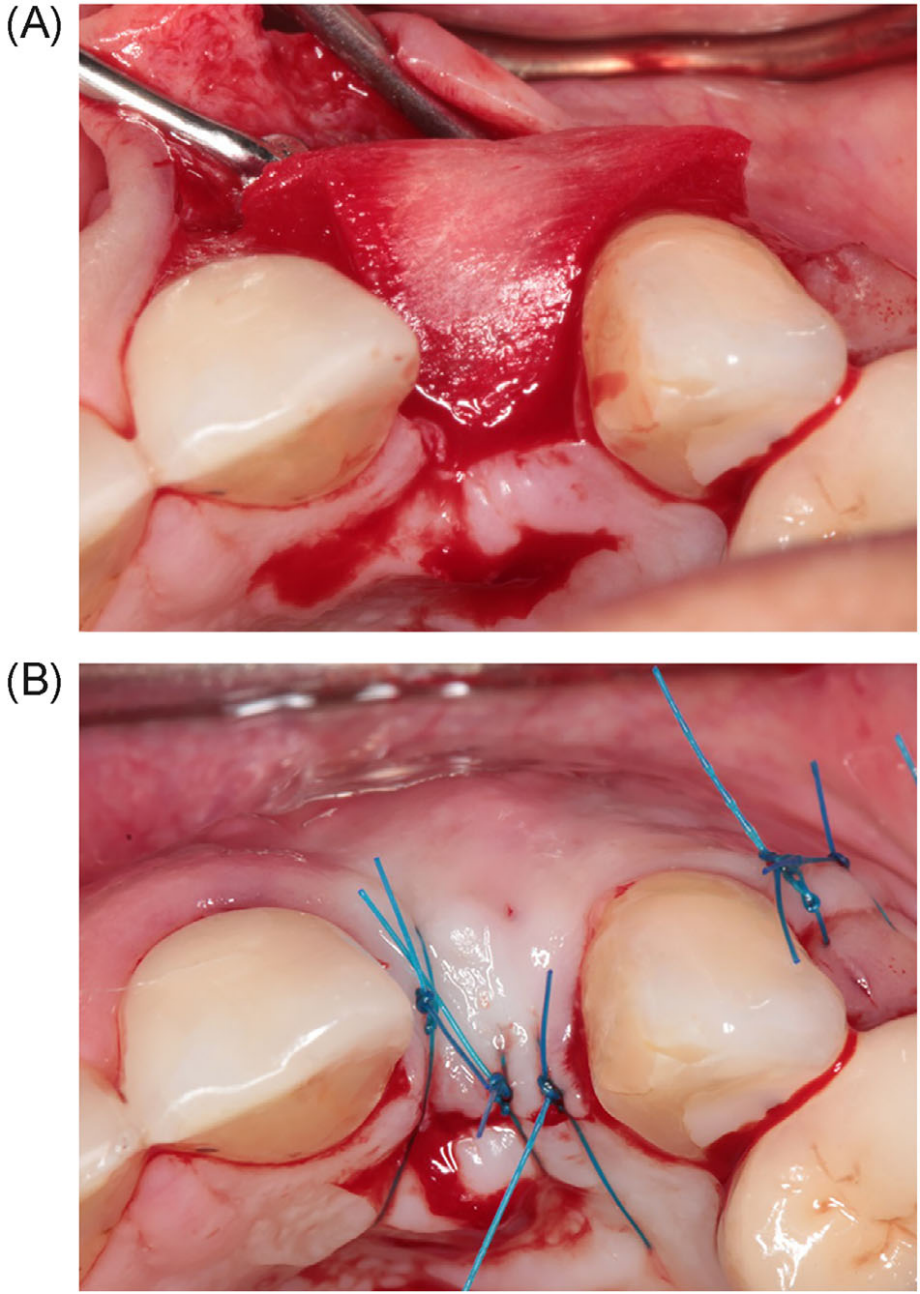
(A) Placement of the XCM in the prepared mucoperiosteal envelope. (B) XCM secured with horizontal and vertical mattress sutures. XCM, xenogeneic collagen matrix.

The implant was inserted 4 months after the augmentation procedure. A small palatal crest‐incision was made to expose the alveolar ridge, followed by extensions through the buccal and palatal sulcus of the adjacent teeth and a divergent relieving incision at the distal tooth to elevate the minimal mucoperiosteal flap. The implant site was prepared and guided with a surgical template representing the ideal position of the prospective implant crown. All implants† were installed with a torque controller with 45 Ncm and provided with a cover screw. The implant shoulder was placed 3 mm apical to most facial and cervical aspects of the prospective clinical crown to ensure a proper emergence profile, including being leveled with the alveolar bone. The randomization procedure was done immediately after implant installation. Regarding the CTG group, the CTG was harvested from the palate. A mucoperiosteal envelope was prepared at the facial site and either a CTG or an XCM‡ (XCM group) was placed in the envelope. Graft and matrix were secured with 4–0 acrylic vertical and horizontal mattress sutures (Figures 1A,B and 2A,B).§ In the control group no graft or matrix was placed. In all groups, the wound at the implant site was closed with 5–0 nylon sutures‖. All sutures were removed 2 weeks after surgery. During the healing phase, patients wore a removable partial denture that did not interfere with the wound.

After 3 months, second‐stage surgery took place and a provisional crown was placed on the implant. After 3 months with the provisional crown, a definitive crown was placed with an individualized zirconia base.¶

### 2.3 Outcome measures

The change in mid‐buccal mucosa level (MBML) was assessed on photographs (primary outcome). The secondary outcome measures were a change in interproximal mucosa level, radiographic marginal bone level proximal to the implant, papilla volume, peri‐implant mucosa health, bleeding upon probing, probing pocket depth, aesthetics, and patients’ satisfaction.

### 2.4 Photographic assessment

MBML and inter‐proximal mucosal levels (IML) were assessed before tooth extraction (T_pre_), 1 month (T_1_), and 60 months (T_60_) after final implant crown placement. Pink Esthetic Score‐White Esthetic Score (PES/WES) was assessed from photographs taken at T_60_.^25^

### 2.5 Radiographic assessment

Marginal bone level was measured at T_60_ and compared with the level at T_1._ ^26^

### 2.6 Clinical assessment

Clinical data of any implant was collected by a single examiner (E.G.Z.), who was blinded regarding group allocation, at T_1_ and T_60_. Clinical data were collected at T_1_ and T_60_. The following parameters were assessed: (1) volume of the interproximal papilla (Papilla index),^27^ peri‐implant mucosa health (Gingival‐index),^28^ (3) bleeding upon probing (Modified sulcus bleeding index),^29^ and probing pocket depth.

### 2.7 Patient satisfaction

An OHIP‐14 (Oral Health Impacts Profile) questionnaire^30^ and specific questions about satisfaction with the implant and crown to be answered on a 10cm Visual Analogue Scale (VAS), were completed at T_pre_ and T_60_.

### 2.8 Statistical analysis

The sample size was calculated based on a clinically relevant difference in the recession of the mid‐buccal mucosa of 0.5 ± 0.6 mm from implant placement to 1 year after the final implant crown placement between groups, a power of 80%, and an alpha of 5%. This resulted in a minimum sample size of 18 patients per group. To account for the loss to follow‐up, 20 patients per group were included.

The nominal and ordinal outcome measures were presented as frequencies and corresponding percentages. The distribution of the continuous variables was checked visually using histograms and QQ plots as well as using the Shapiro–Wilk test. Normally distributed continuous data were described as mean and standard deviation (SD). Skewed distributed continuous variables were presented as median with interquartile range (P25–P75). Nominal and ordinal outcome measures were compared between groups using Fisher's exact test. Normally distributed continuous data were compared using the 1‐way analysis of variance (ANOVA) test. Skewed distributed continuous variables were compared using the Kruskal–Wallis test. In case of significant differences between groups, a post‐hoc test using Bonferroni's correction was performed. Paired samples *t*‐test was performed for within person comparison of normally distributed data. A significance level of *p* < 0.05 was chosen for all the analyses. The statistical analyses were performed in R, version 4.0.5 (R Core team), using the stats package.

## 3 RESULTS

**FIGURE 3:**
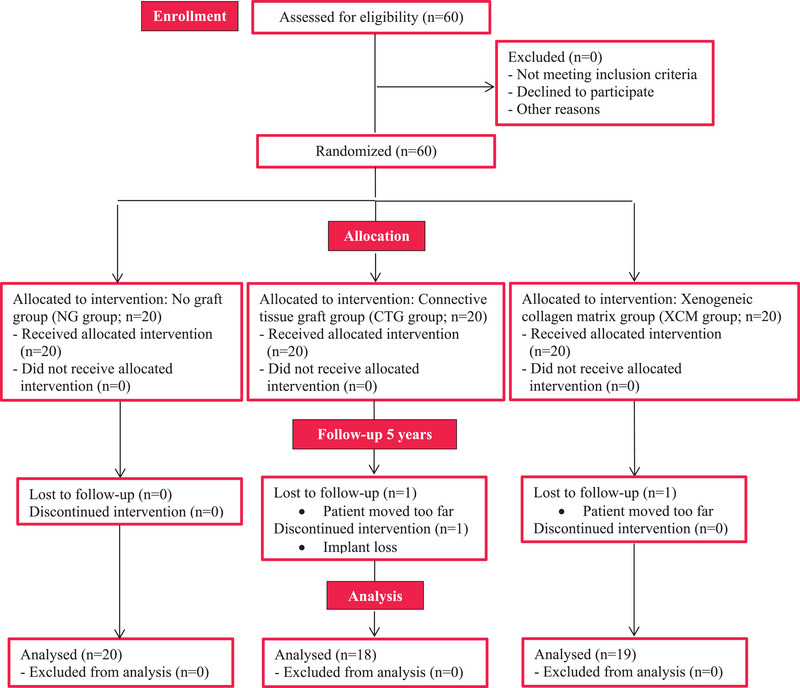
CONSORT flow diagram.

Baseline patient characteristics are depicted in Table 1. At the 5‐year follow‐up, all patients of the NG group could be evaluated without any discontinued intervention. Of the CTG group, 1 patient moved too far to attend the evaluation and 1 patient lost the implant. Of the XCM group, 1 patient moved too far to attend the evaluation (Figure 3).

**TABLE 1:** Patient characteristics per study group.

Variable	NG group (*n* = 20)	CTG group (*n* = 20)	XCM group (*n* = 20)
Male/female (*n*)	7/13	11/9	7/13
Mean age ± SD in years; (range)	42.0 ± 15.7 (18–71)	38.2 ± 16.7 (18–69)	45.4 ± 17.0 (18–73)
Implant site location I1/I2/C/P1	12/8/0/0	16/3/1/0	11/4/3/2

Abbreviations: CTG, connective tissue graft; NG, no graft; SD, standard deviation; XCM, xenogeneic collagen matrix.

### 3.1 Change in MBML

**FIGURE 4:**
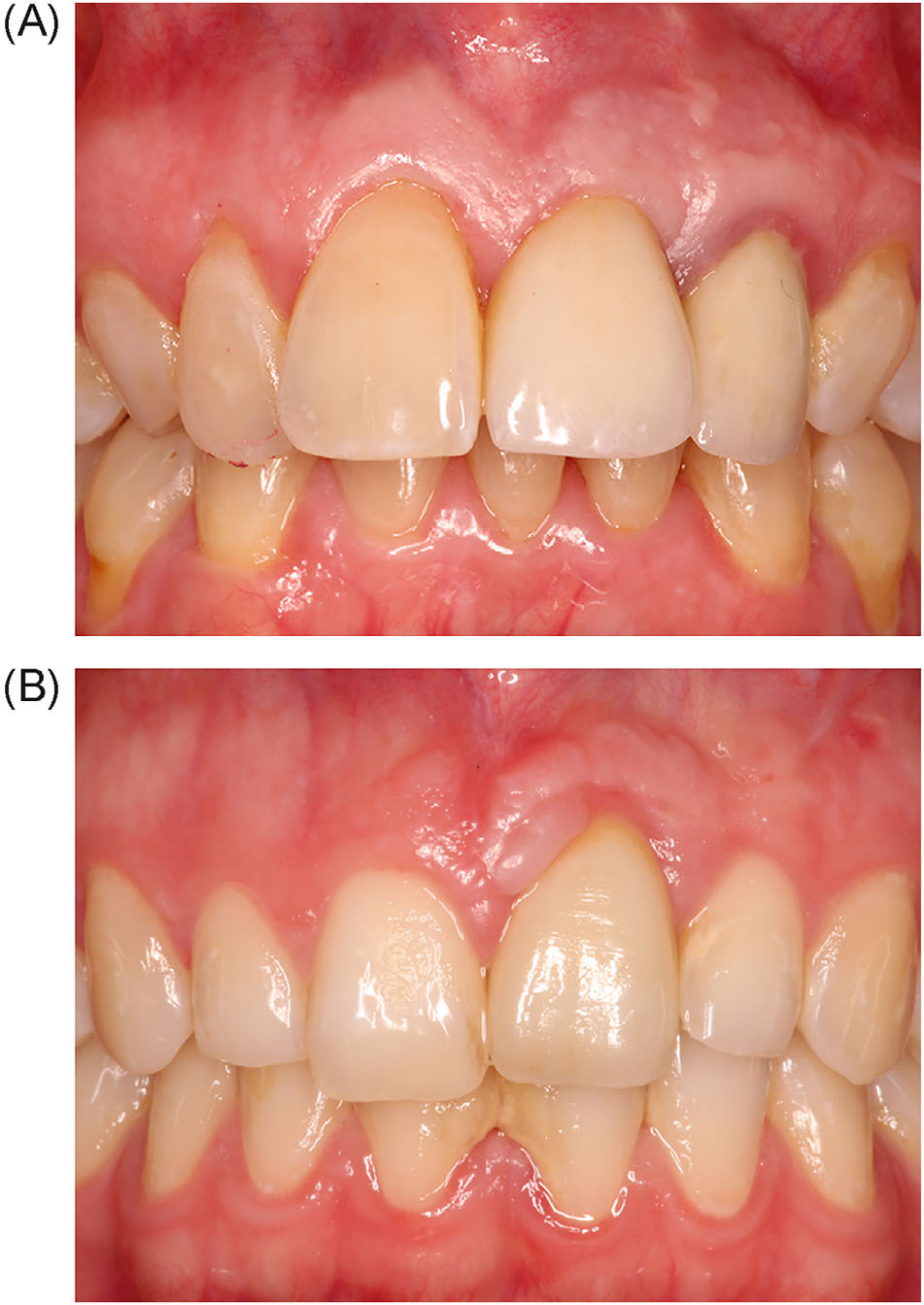
(A) Example of a good result in the NG group. (B) Example of a bad result in the NG group. NG, no graft.

**FIGURE 5:**
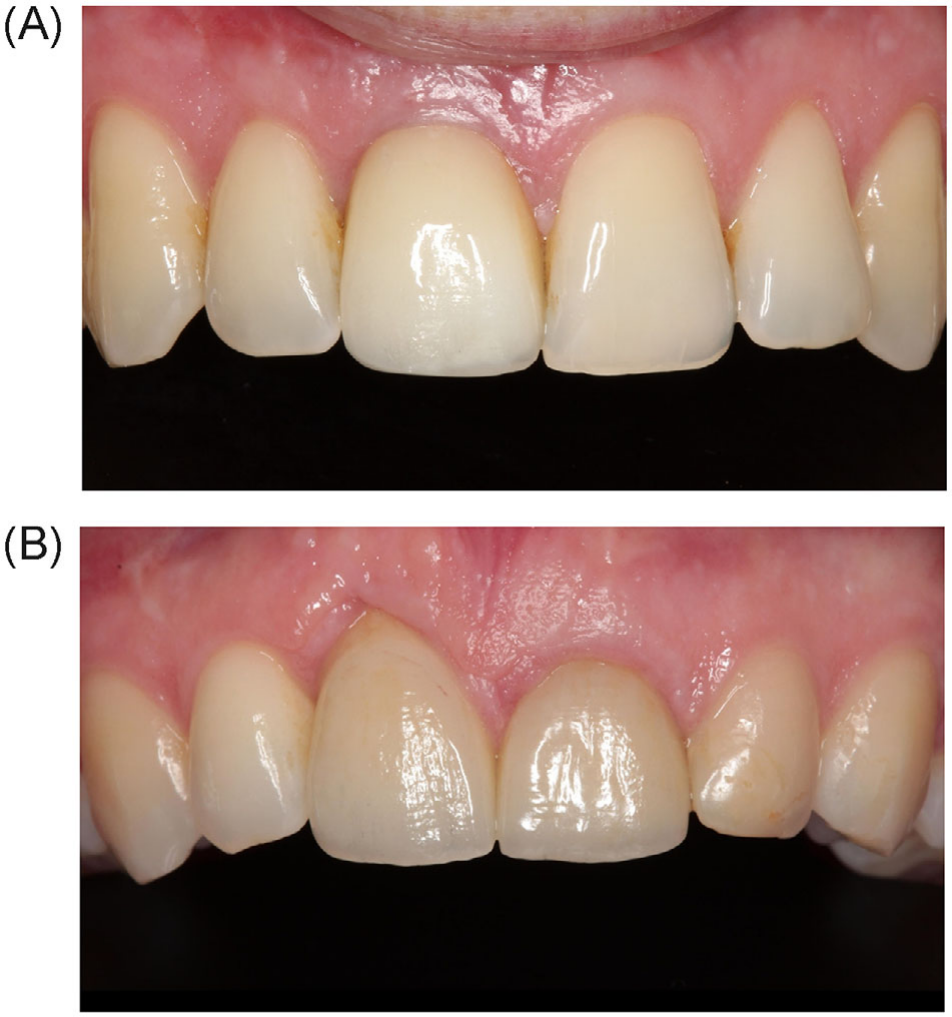
(A) Example of a good result in the CTG group. (B) Example of a bad result in the CTG group. CTG, connective tissue graft.

**FIGURE 6:**
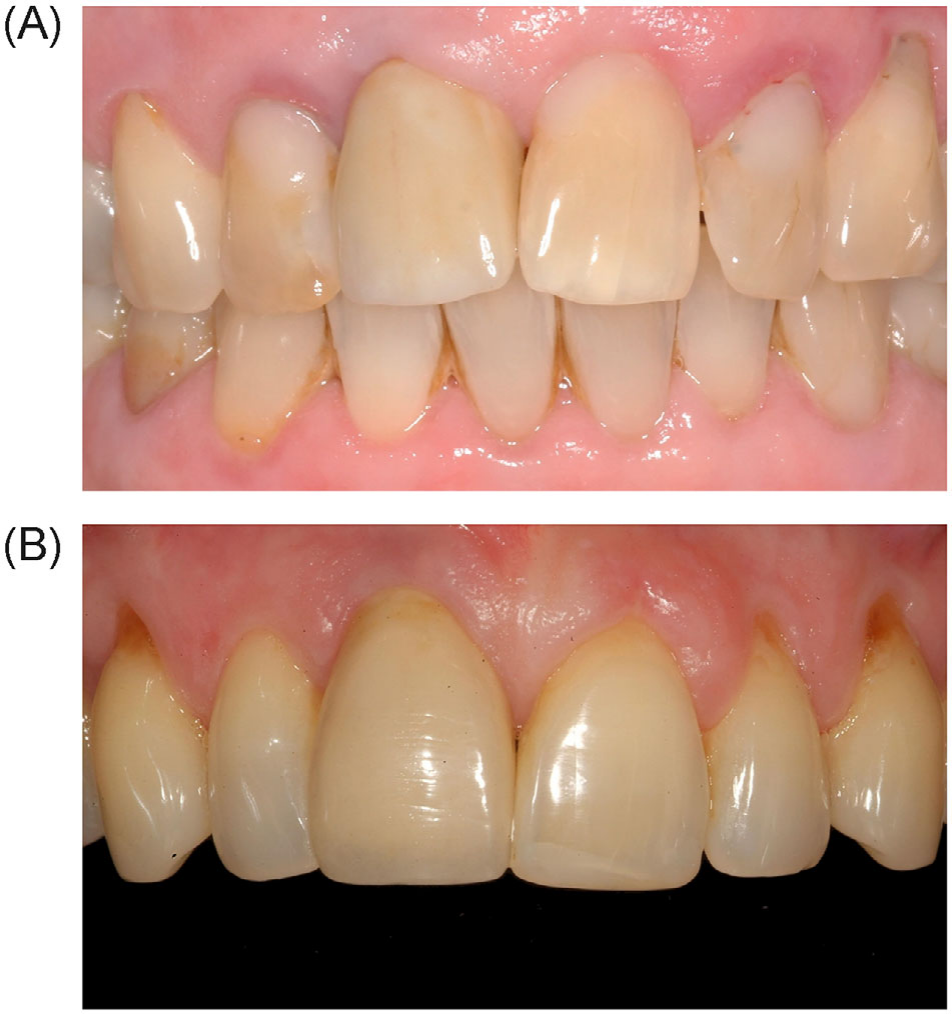
(A) Example of a good result in the XCM group. (B) Example of a bad result in the XCM group. XCM, xenogeneic collagen matrix.

Change in MBML was limited in all 3 groups throughout the evaluation period and no significant differences between the groups were observed (Table 2). At T_60_, the MBML showed an average recession, compared to the preoperative situation, of 0.61 ± 1.72 mm in the NG group, 0.41 ± 1.20 mm in the CTG group, and 0.30 ± 1.22 mm in the XCM group (*p* = 0.78). Examples of good and bad results in the respective groups are given in Figures 4, 5, and 6A,B.

**TABLE 2:** Mean marginal soft tissue level change and marginal bone level change, and papilla volume (%) from baseline (T_pre_), from 1 month after definitive restoration placement (T_1_) to 5 years after definitive restoration placement (T_60_).

	T_pre_–T_60_				T_1_–T_60_			
	NG group	CTG group	XCM group		NG group	CTG group	XCM group	
Parameter	*n* = 20	*n* = 18	*n* = 19	*p*‐value	*n* = 20	*n* = 18	*n* = 19	*p*‐value
Mid‐buccal mucosa level change	−0.61 (1.72)	−0.41 (1.20)	−0.30 (1.22)	0.78	−0.27 (0.70)	−0.37 (0.45)	−0.32 (0.45)	0.86
*Interproximal mucosa level change*								
Mesial side of implant	−1.02 (1.29)	−0.89 (0.83)	−0.47 (0.85)	0.23	−0.11 (0.77)	−0.06 (0.50)	−0.07 (0.80)	0.98
Distal side of implant	−0.90 (0.99)	−0.89 (0.95)	−0.94 (0.71)	0.99	−0.04 (0.82)	−0.01 (0.68)	−0.09 (0.92)	0.87
*Marginal bone level change*								
Mesial side of implant	NA	NA	NA	NA	−0.14 (0.81)	−0.21 (0.66)	0.09 (1.14)	0.56
Distal side of implant	NA	NA	NA	NA	0.01 (1.10)	0.00 (0.59)	0.01 (0.63)	>0.99

*Papilla volume* (mesial side/distal side)	T_pre_				T_60_			
	*n* = 20	*n* = 20	*n* = 20	0.55/0.68	*n* = 20	*n* = 18	*n* = 19	0.23/0.99
Score 0	0%/5%	0%/0%	5%/5%		0%/0%	0%/0%	0%/0%	
Score 1	65%/45%	55%/50%	35%/40%		5%/5%	6%/6%	6%/10%	
Score 2	25%/40%	35%/50%	40%/55%		55%/55%	22%/50%	47%/53%	
Score 3	10%/10%	10%/0%	20%/0%		40%/40%	72%/44%	47%/37%	
Score 4	0%/0%	0%/0%	0%/0%		0%/0%	0%/0%	0%/0%	

Abbreviations: CTG, connective tissue graft; NG, no graft; XCM, xenogeneic collagen matrix.

### 3.2 Change in radiographic marginal bone level

Between T_1_ and T_60_ mean marginal bone level changes were −0.14 ± 0.81 for the mesial side and 0.01 ± 1.10 for the distal side in the NG group, respectively. For the CTG group changes were −0.21 ± 0.66 and 0.00 ± 0.59 and for the XCM group changes were 0.09 ± 1.14 and 0.01 ± 0.63, respectively. Differences were not significant between the groups (mesial side: *p* = 0.56, distal side: *p* > 0.99; Table 2).

### 3.3 Clinical outcome

Papilla volume around the implant crown at T_60_ is depicted in Table 2. In all groups, papilla volume is mostly at least half of the papilla is present to the presence of a full papilla (p_mesial_ = 0.23 and p_distal_ = 0.99). The low numbers on the Gingiva‐index and the Bleeding‐index show that there is good peri‐implant health as well after 1 month as after 5 years of functioning (without a significant difference between the groups) (Table 3). Mean pocket probing depths were in general below 3 mm without a significant difference between the groups (Table 3).

**TABLE 3:** Peri‐implant health at 1 month (T_1_) and 60 months (T_60_) after definitive restoration placement.

	T_1_				T_60_			
	NG group	CTG group	XCM group		NG group	CTG group	XCM group	
Parameter	*n* = 20	*n* = 20	*n* = 20	*p*‐value	*n* = 20	*n* = 18	*n* = 19	*p*‐value
Gingiva‐index (median with IQR)	0 (0–0)	0 (0–0)	0 (0–0)	0.37	0 (0–0)	0 (0–0)	0 (0–0)	0.27
Bleeding‐index (median with IQR)	1 (0–1)	0 (0–1)	1 (0–1)	0.28	0 (0–0)	0 (0–1)	1 (0–1)	0.65
*Probing depth (mean* ± *SD)*								
Mesial side of implant	2.5 (1.1)	2.4 (1.3)	2.8 (1.2)	0.64	2.3 (1.0)	2.5 (1.2)	2.4 (1.0)	0.84
Mid‐buccal of implant	2.7 (1.2)	3.2 (1.2)	2.8 (1.6)	0.44	2.2 (0.8)	2.5 (1.4)	2.1 (0.9)	0.48
Distal side of implant	2.3 (0.6)	2.7 (1.0)	2.9 (0.9)	0.06	2.6 (0.9)	2.4 (1.4)	2.5 (1.0)	0.96
Palatal of implant	2.0 (0.8)	2.5 (0.7)	2.6 (0.8)	0.06	1.9 (0.6)	1.9 (0.8)	2.1 (0.9)	0.62

Abbreviations: CTG, connective tissue graft; IQR, interquartile range; NG, no graft; SD, standard deviation; XCM, xenogeneic collagen matrix.

### 3.4 Esthetic assessment

No significant inter‐group differences were found with respect to PES and WES total scores (Table 4). Acceptable levels of esthetics were reached in all groups. However, it must be said that mucosa was rated less than the implant crown by the professional.

**TABLE 4:** Patient satisfaction and professional opinion (mean ± SD) at baseline (T_pre_) and 60 months after definitive restoration placement (T_60_).

	T_pre_				T_60_			
	NG group	CTG group	XCM group		NG group	CTG group	XCM group	
Parameter	*n* = 20	*n* = 20	*n* = 20	*p*‐value	*n* = 20	*n* = 18	*n* = 19	*p*‐value
*Patient's opinion*								
VAS questions (0–10)								
How satisfied are you with the implant and the implant crown in general?	NA	NA	NA	NA	9.1 (0.8)	8.6 (1.4)	9.2 (1.1)	0.31
Color of the crown	NA	NA	NA	NA	9.2 (0.9)	7.9 (2.5)	8.7 (1.8)	0.09
Form of the crown	NA	NA	NA	NA	9.4 (0.8)	8.3 (2.4)	9.3 (1.0)	0.08
Color of the peri‐implant mucosa	NA	NA	NA	NA	8.8 (1.1)	7.5 (2.7)	9.1 (1.2)	0.17
Form of the peri‐implant mucosa	NA	NA	NA	NA	7.9 (2.2)	7.4 (2.7)	8.5 (1.6)	0.31
Total OHIP‐score (0–70)	29.7 (11.1)	31.9 (8.2)	29.6 (10.1)	0.71	15.3 (2.5)	17.4 (4.5)	18.3 (3.6)	0.03
*Professional's opinion*								
PES total (0–10)	NA	NA	NA	NA	6.2 (1.6)	6.2 (1.6)	5.7 (1.3)	0.49
WES total (0–10)	NA	NA	NA	NA	7.5 (1.3)	7.8 (1.3)	7.4 (1.6)	0.62
PES/WES total (0–20)	NA	NA	NA	NA	13.6 (2.3)	14.1 (2.1)	13.1 (2.6)	0.47

Abbreviations: CTG, connective tissue graft; NA, not applicable; NG, no graft; OHIP, Oral Health Impacts Profile; PES, Pink Esthetic Score; SD, standard deviation; VAS, Visual Analog Scale; WES, White Esthetic Score; XCM, xenogeneic collagen matrix.

### 3.5 Patient satisfaction

At T_60_, the results of the VAS scores showed no difference in overall patient satisfaction (*p* = 0.31), and was judged as high (Table 4). By patients, the form of the mucosa received the lowest scores. An inter‐group difference was found for the total OHIP questionnaire scores. The mean score at T_60_ in the NG group was better if compared with the XCM group (*p* = 0.03). Within‐group comparisons showed a favorable and significant improvement between T_pre_ and T_60_ for all 3 treatment groups (*p* < 0.001)).

## 4 DISCUSSION

In this 5‐year RCT, the use of a CTG or an XCM did not result in better retention of the level of the mid‐buccal mucosa and a better esthetic outcome compared to the use of no soft tissue graft if applied at the time of implant placement after alveolar ridge preservation with mucosa grafts from the maxillary tuberosity. The observed recession of the mid‐buccal mucosa between the time of the failing tooth still present and 5 years after definitive restoration placement was minor in all 3 groups and within clinically acceptable levels. Furthermore, only a limited change was found between definitive restoration placement and 5 years. These limited changes are said to be clinically acceptable.^31^

Results of the present study can best be compared with the medium‐term studies on soft tissue augmentation procedures of Cosyn et al.,^19^ Fenner et al.,^20^ Hosseini et al.,^21^ Thoma et al.,^22^ and Zuiderveld et al.^23^ In all studies, it was reported that recession was very limited in groups with and without soft tissue augmentation. Only in the study of Zuiderveld et al.^23^ a significant difference was found, with less recession in the CTG group. Thoma et al.^22^ reported on the 5‐year comparison between the application of a CTG and an XCM and also in this study changes were small without a significant difference between the procedures. Results in these studies are in line with the present study, reporting that the effect of the application of a soft tissue augmentation procedure on the MBML is limited. This might be explained by the fact that the extraction sockets after removing the tooth were sealed with a mucosa graft, which is beneficial in preserving the soft tissue contour.^32^, ^33^ It could be that this initial soft tissue augmentation technique in all 3 groups contributed to the preservation of sufficient peri‐implant soft tissue, leading to no further effect of additional soft tissue at implant placement.

Changes in marginal bone level were limited after 5 years in this study without a significant difference between the groups. Also, Fenner et al.,^20^ Thoma et al.,^22^ and Zuiderveld et al.^23^ reported limited changes in all groups. However, Zuiderveld et al.^23^ reported a significant difference in bone level change on 1 side of the implant in favor of the NG group. In general, it can be said that the effect of a soft tissue augmentation is limited on marginal bone level changes during a 5‐years follow‐up.

Peri‐implant soft tissue health was evaluated with the use of the Gingiva‐index, the Bleeding‐index, and pocket probing depth in the present study. Cosyn et al.^19^ did not report detailed information about the group that underwent a soft tissue augmentation. Hosseini et al.^21^ and Thoma et al.^22^ did not report soft tissue health outcomes. Fenner et al.^20^ and Zuiderveld et al.^23^ reported healthy soft tissues and did not find a significant difference between the group with a CTG and the group without a CTG. Outcomes in the latter 2 studies are in line with the healthy peri‐implant soft tissue health outcomes in the present study without a significant difference between groups.

There was not a significant difference in Pink Esthetic Scores (PES) scores between the groups. Also, Thoma et al.^22^ and Zuiderveld et al.^23^ evaluated PES and concluded that there were no differences between different soft tissue augmentation procedures. Apparently, after 5 years an augmentation procedure (whether a CTG or an XCM) did not enhance the esthetics of the peri‐implant mucosa, neither did the extra surgical procedure worsen the situation. Mean PES scores of respectively 6.6, 7.0, and 6.1 in the NG group, CTG group, and XCM group are not of the highest level. In all cases, at removing the maxillary single failing tooth there was a vertical buccal bone wall defect of >5 mm of the extraction socket. Therefore, all extraction sockets were augmented prior to implant insertion and closed with a mucosa graft. This extra bone augmentation procedure and mucosa graft procedure to fill and close the extraction socket could have influenced the final soft tissue result. Thoma et al.^22^ and Zuiderveld et al.^23^ evaluated patient satisfaction. Results were in line with the present study revealing very satisfied patients without any difference between the groups.

It must be noted that the timing of soft tissue augmentation surgery varied in the compared studies. In the present study the application of soft tissue was done at the time of implant placement in preserved implant sites. In the study of Cosyn et al.^19^ the CTG was done 3 months after implant placement and connection of a provisional restoration in immediate implant sites. In the study of Fenner et al.^20^ the CTG was placed 2 weeks after implant surgery in healed sites. In the study of Hosseini et al.^21^ the CTG was placed 3 months after implant surgery in healed sites. In the study of Thoma et al.^22^ the soft tissue augmentation was done 3 months after implant placement in healed sites. Zuiderveld et al.^23^ applied the CTG at implant placement and connection of a provisional restoration in immediate implant sites. Next to this, in the study of Cosyn et al.,^19^ Fenner et al.,^20^ Hosseini et al.,^21^ and Thoma et al.^22^ the effect of a soft tissue augmentation procedure was evaluated in cases with a deficiency in mid‐buccal mucosa level or a deficiency in mucoca thickness. Whereas in the study of Zuiderveld et al.^23^ and in the present study the soft tissue augmentation procedure was performed in all patients, whether or not there was a soft tissue deficiency. Notwithstanding these differences in timing and site characteristics, it seems that the effect of soft tissue augmentation is minimal after 5 years. This is in contrast with the systematic review of Raghoebar et al.^13^ in which was concluded that a soft tissue augmentation in the esthetic region results in less recession (compared with NG) following immediate implant placement or delayed implant placement. However, in this systematic review, mainly short‐term studies were included. Perhaps after a longer follow‐up period, the effect of a soft tissue augmentation is diminishing as also was shown in this study.

A limitation of the present study is that the majority of studies on the effect of soft tissue grafting also assess the change in mid‐buccal mucosal volume. Measurement of the change in mid‐buccal mucosal volume would have been desirable but was beyond the scope of this study. Another limitation of the study is the lack of information on peri‐implant bone stability between the time of implant placement and T_1_. Bone level change after implant placement may have affected the soft tissue level and the surgical procedure may have affected bone stability. Intra‐oral radiographs made at the time of implant placement could have given information on possible changes in mesial and distal bone levels between the time of implant placement and T_1_. Cone beam computed tomography made throughout the evaluation period could have given information on the stability of buccal bone thickness.

## 5 CONCLUSION

Within the limitations of this study, it can be concluded that the application of a soft tissue graft combined with placement of a single implant in a preserved alveolar ridge, and with sealing the socket with a mucosa graft, in the esthetic zone did result in stable soft tissue levels during the evaluation period, but did not result in a more favorable esthetic outcome after 5 years than when no soft tissue graft was applied during implant placement.

## AUTHOR CONTRIBUTIONS

All the authors contributed substantially to the conception, design, data interpretation, and critical revision of the study and the manuscript, and approved the final version for publication. Elise G. Zuiderveld and Henny J. A. Meijer were involved in collecting the data and drafting the manuscript. Henny J. A. Meijer and Barzi Gareb were involved in the data analysis.

## CONFLICT OF INTEREST STATEMENT

The authors report no conflicts of interest.

## Data Availability

The data that support the findings of this study are available from the corresponding author upon reasonable request.

## Footnotes

* Bio‐Oss, Geistlich Pharma AG, Wolhusen, Switzerland

† Nobel Replace CC, Nobel Biocare AG, Gothenburg, Sweden

‡ Mucograft, Geistlich Pharma AG, Wolhusen, Switzerland

§ Vicryl, Ethicon, Johnson & Johnson, Amersfoort, The Netherlands

‖ Ethilon, Ethicon, Johnson & Johnson

¶ NobelProcera, Nobel Biocare AG, Gothenburg, Sweden
